# Supplementary material for: Will Africans take COVID-19 vaccination?
Source: PLoS One. 2021 Dec 1;16(12):e0260575. doi: 10.1371/journal.pone.0260575 (PMC8635331; doi:10.1371/journal.pone.0260575)
Supplement: S1 File — (DOCX) [file pone.0260575.s001.docx]

## **Supporting information**

## **Supplement 1. Research Questionnaire**

**Perception and Willingness to accept COVID-19 Vaccine in Africa**

1. Age………………….18-24 (1); 25-34 (2); 35-44 (3); 45-54 (4); 55-64 (5); >65 (6)

2. Gender (M/F). Male (1) Female (2)

3. Highest Level of Education Completed

a. None (0) Primary School (1) Secondary school (2) OND/technical degree (3) University Degree (undergraduate) (4) Graduate Degree (5)

4. Country of Residence…………… State/ region/ district…………….

Community setting (Urban/semi-urban/rural) Rural (1) Semi-Urban (2) Urban (3)

1. Dropdown menu to choose state.

5. Are you employed? (Y/N) Yes (1) No (0)

6. If yes to #5, are you employed in the healthcare field? (Y/N). Yes (1) No (0)

7. What is your household income per month (or year in dollars or local currency)?

(Could have 5 options to select from). $ 99 (1) Between $100- $499 (2) Between $500-$999 (3) Between $1000-$4999 (4) Between $5000-$9999 (5) Between $10,000-$14,999 (6) Above $15,000 (7)

9. What religion do you identify with?

a.  Christianity (1) Islam (2) Traditional Religion (3) Others (4) None (0)

10.  Marital status?

Single (1) Married (2) Widow(er) (3) cohabiting (4)

Dropdown menu to choose marital status.

11.  Are you a parent? (Y/N). Yes (1) No (0).

12.  Do you have a medical condition that prevents you from getting vaccinations? (Y/N) Yes (1) No (0)

**Survey questions**

*(Most are Likert scale;* *Strongly disagree, disagree, neutral, agree, strongly agree)*

**Self-reported health status and ‘health literacy’**

1. If yes above, please state

2. I get most of my information about health from:

a. -Religious leaders (1), community elders (2), NGO (3), govt (4), healthcare workers (5), news media (6), social media (7), celebrities (8), schools (9), public health organizations (10).

3. I am comfortable using this information to make decisions about my health. Yes (1), No (0).

4. I am the one who decides whether my child receives vaccines. No (0), Yes (1), Not applicable (2).

**Knowledge & perception of vaccines**

6. I understand how vaccines work. No (0), Yes (1).

7. I am aware that there are routine vaccinations that are recommended for children No (0), Yes (1)

8. I am aware that some vaccinations are recommended for adults. No (0), Yes (1).

9. I believe that vaccines can prevent serious infectious diseases. Strongly disagree (0), disagree (1), Indifferent (2), agree (3), strongly agree (4).

10.  I think it is important for everyone to get recommended vaccinations. Strongly disagree (0), disagree (1), Indifferent (2), agree (3), strongly agree (4).

11.  I believe my community is better protected from a disease if most people are vaccinated. Strongly disagree (0), disagree (1), Indifferent (2), agree (3), strongly agree (4).

12.  I believe that most people tolerate vaccinations very well. Strongly disagree (0), disagree (1), Indifferent (2), agree (3), strongly agree (4).

13.  I believe that the risks of vaccination are more than the benefits. Strongly disagree (0), disagree (1), Indifferent (2), agree (3), strongly agree (4).

**Past experiences/ behaviours**

15.  I know someone who has gotten a vaccine-preventable disease because they did not get the vaccine. No (0), Yes (1).

16.  I know someone who has had a serious side effect from a vaccination. No (0), Yes (1).

17.  In the past I have been advised not to give my child a recommended vaccine. No (0), Yes (1), NA (2).

a. If yes, who gave the advice? (Religious leaders (1), community elders (2), healthcare workers (3), family member (4), spouse (5), friend (6), Not applicable (7)).

18.  In the past, I have refused a vaccine that was recommended for me or my child. No (0), Yes (1), Not applicable (2).

19.  In the past, I have done my best to get all the recommended vaccines for me or my child. No (0), Yes (1), Not applicable (2).

20.  In the past, I have not been able to get a vaccine that I planned to get. No (0), Yes (1).

a. If yes, why: (distance to health center (1), did not have time (2), unable to afford (3), vaccine not available (4), other (5)).

**Knowledge and risk assessment of SARS-COV-2**

21.  I know a family member or friend who has been sick with coronavirus. No (0), Yes (1).

22.  I believe my risk of becoming infected with coronavirus is (very low (1), low (2), medium (3), high (4), very high (5))

23.  I believe my risk of falling very sick IF I get infected with coronavirus is (very low (1), low (2), medium (3), high (4), very high (5)).

**Acceptance of COVID-19 vaccine**

25.  If there was a vaccine available to prevent coronavirus, I would want to get it as soon as possible. No (0), Yes (1).

26.  If there was a vaccine available to prevent coronavirus, I would wait and see how other people respond to it before I get it. No (0), Yes (1).

27.  I would be willing to participate in a clinical trial for a coronavirus vaccine. No (0), Yes (1).

28.  I do not think a coronavirus vaccine is necessary. No (0), Yes (1).

29.  I believe that there are other (better) ways to protect against coronavirus than a vaccine. No (0), Yes (1).

30.  I am most likely to get the coronavirus vaccine if it is recommended by

a. Religious leaders (1), community elders (2), NGO (3), govt (4), healthcare workers (5), scientists (6), news media (7), social media (8), celebrities (9), schools (10), public health organizations (NCDC) (11), my own research (12), friends (13), family (14), Not applicable (15).

31.  I am most likely to take the vaccine if it is in the form of

a. (Injection (1), oral (taken by mouth) (2), nasal spray (spray into nose) (3), no preference (4)).

32.   I am worried about the possible side effects of the coronavirus vaccine. No (0), Yes (1).

33.  I worry that I can get infected with coronavirus by getting the vaccine. No (0), Yes (1).

34.   I worry that people are using the coronavirus vaccine as an excuse to ‘experiment’ on Africans. Strongly disagree (0), disagree (1), Don’t know (2), agree (3), strongly agree (4).

35.   I worry that the coronavirus vaccine will not actually work to prevent COVID-19. Strongly disagree (0), disagree (1), Don’t know (2), agree (3), strongly agree (4).

**Voluntariness and affordability**

36.  If there is a vaccine available for coronavirus, I believe it should be mandatory. Strongly disagree (0), disagree (1), Don’t know (2), agree (3), strongly agree (4).

37.  I will ONLY get the coronavirus vaccine if it is mandatory. No (0), Yes (1).

38.  I will get the coronavirus vaccine even if it is NOT mandatory. No (0), Yes (1).

39.  If there is a vaccine available for coronavirus, I believe it should be free. Strongly disagree (0), disagree (1), Neutral (2), agree (3), strongly agree (4).

40.   I consider [----] to be a reasonable price range for the coronavirus vaccine

a. (Options:1-3 dollars (1), 4-6 dollars (2), 7-9 dollars (3), =/>10 dollars (4)).

**Vaccine education & distribution**

  42.  I am most likely to trust information about vaccines from.

a. None (0), Religious leaders (1), community elders (2), NGO (3), govt (4), healthcare workers (5), scientists (6), news media (7), social media (8), celebrities (9), schools (10), public health organizations (NCDC) (11), my own research (12), friends (13), family (14), Not applicable (15).

43.  It is important for me to know about the risks as well as the benefits of any vaccine. Strongly disagree (0), disagree (1), Neutral (2), agree (3), strongly agree (4).

44.  I am most likely to get the coronavirus vaccine if it is recommended by

a. None (0), Religious leaders (1), community elders (2), NGO (3), govt (4), healthcare workers (5), scientists (6), news media (7), social media (8), celebrities (9), schools (10), public health organizations (NCDC) (11), my own research (12), friends (13), family (14), Not applicable (15).

45.  To reach my nearest health center, it normally takes

a. <15min (1), <30min (2), 1hr (3), <2hr (4), >2hr (5).

46.  I would be willing to travel up to […] to get the coronavirus vaccine

a. <15min (1), <30min (2), 1hr (3), <2hr (4), >2hr (5).

47.  I would prefer community workers to come to my house or place of work to give the coronavirus vaccine, rather than me going to a health center. Strongly disagree (0), disagree (1), Neutral (2), agree (3), strongly agree (4).

48.  What type of COVID-19 vaccine do you know (Brand/Company)? Moderna (1), Barda (2), Niaid (3), Astrazeneca (4), OWS (5).

## **S1 Table. Vaccine Acceptance and hesitancy in different African countries**

| COVID-19 Vaccine Acceptability | | | |
| --- | --- | --- | --- |
| Countries | Accept | Hesitancy | Total |
| Nigeria | 678 | 353 | 1031 |
| Egypt | 408 | 217 | 625 |
| South Africa | 390 | 130 | 520 |
| Ghana | 269 | 174 | 443 |
| Cameroon | 135 | 274 | 409 |
| Sudan | 302 | 117 | 419 |
| Democratic Republic of Congo | 187 | 210 | 397 |
| Rwanda | 205 | 116 | 321 |
| Morocco | 210 | 107 | 317 |
| Kenya | 88 | 61 | 149 |
| Tanzania | 61 | 25 | 86 |
| Liberia | 65 | 12 | 77 |
| Malawi | 28 | 10 | 38 |
| Other African Coutries | 36 | 19 | 55 |
| Africans in Diaspora | 215 | 109 | 324 |
| Overall | 3277 | 1934 | 5211 |

## **S2 Table. Self-reported Health Status and Health Literacy**

| **Question** | **Yes, n (%)** | **No, n (%)** | **Not applicable** |
| --- | --- | --- | --- |
| Do you have a medical condition that prevents you from getting vaccinations? | 543 (10.4) | 4670 (89.6) | - |
| I am comfortable using this information to make decisions about my health | 4818 (92.4) | 394 (7.6) | - |
| I am the one who decides whether my child receives vaccines | 2861 (54.9) | 479 (9.2) | 1872 (35.9) |

## **S3 Table. Source (s) of personal information on health matters (n = 5212)**

| **Sources of information^[[1]](#footnote-1)^** | **Yes (%)** | **No (%)** |
| --- | --- | --- |
| Religious Leaders | 275 (5.3) | 4937 (94.7) |
| Community Leaders | 178 (3.4) | 5034 (96.6) |
| NGO | 484 (9.3) | 4728 (90.7) |
| Government | 1516 (29.1) | 3696 (70.9) |
| Healthcare workers | 2639 (50.6) | 2573 (49.4) |
| Scientist | 2275 (43.6) | 2937 (56.4) |
| News media | 2249 (43.2) | 2963 (56.8) |
| Social media | 57 (1.1) | 5155 (98.9) |
| Celebrities | 597 (11.5) | 4615 (88.5) |
| Schools | 2121 (40.7) | 3091 (59.3) |
| Research/Journals/Medical Sources | 100 (1.9) | 5112 (98.1) |
| Others | 65 (1.2) | 5147 (98.8) |
| Internet/Google | 22 (0.4) | 5190 (99.6) |

## **S4 Table. Knowledge and perception of vaccines**

| **Variables** | **Yes (%)** | **No (%)** |  | | |
| --- | --- | --- | --- | --- | --- |
| I understand how vaccines work | 4052 (77.7) | 1160 (22.3) |  |  |  |
| I am aware that there are routine vaccinations that are recommended for children | 4679 (89.8) | 533 (10.2) |  |  |  |
| I am aware that some vaccinations are recommended for adults | 4579 (87.9) | 633 (12.2) |  |  |  |
|  | Strongly disagree | Disagree | Indifferent | Agree | Strongly agree |
| I believe that vaccines can prevent serious infectious diseases | 259 (5.0) | 155 (3.0) | 553 (10.6) | 2345 (45.0) | 1900 (36.5) |
| I think it is important for everyone to get recommended vaccinations | 215 (4.1) | 325 (6.2) | 736 (14.1) | 2233 (42.8) | 1703 (32.7) |
| I believe my community is better protected from a disease if most people are vaccinated | 241 (4.6) | 248 (4.8) | 687 (13.2) | 2366 (45.4) | 1670 (32.0) |
| I believe that most people tolerate vaccinations very well | 202 (3.9) | 756 (14.5) | 1315 (25.2) | 2369 (45.5) | 570 (10.9) |
| I believe that the risks of Vaccination are more than the benefits | 870 (16.7) | 2064 (39.6) | 1184 (22.7) | 814 (15.6) | 280 (5.4) |
| I would prefer community and health workers to come to my house or place of work to give the COVID-19 vaccine*. | 557 (10.7) | 710 (13.6) | 1588 (30.5) | 1339 (25.7) | 950 (18.2) |
| Distance as a motivation for vaccination* |  |  |  |  |  |
| Time to reach health center | <15min | <30min | 1hr | <2hr | >2hr |
| To reach the nearest health center, it normally takes | 2234 (42.9) | 1548 (29.7) | 998 (19.1) | 213 (4.1) | 151 (2.9) |
| I would be willing to travel up to […] to get the corona virus (SARS CoV 2) vaccine | 1449 (27.8) | 1279 (24.5) | 1413 (27.1) | 338 (6.5) | 665 (12.8) |

**68 persons did not respond to these questions, which account to 1.3% of the responses.*

## **S5 Table. Reasons for not getting a planned vaccine in the past which may affect getting COVID-19 vaccine (n=1355)**

| Reason | Yes (%) | No (%) |
| --- | --- | --- |
| Distance to health center | 193 (14.2) | 1162 (85.8) |
| Did not have time | 268 (19.8) | 1087 (80.2) |
| Unable to afford | 307 (22.7) | 1048 (77.3) |
| Vaccine not available | 476 (35.1) | 879 (64.9) |
| Other | 85 (6.3) | 1270 (93.7) |


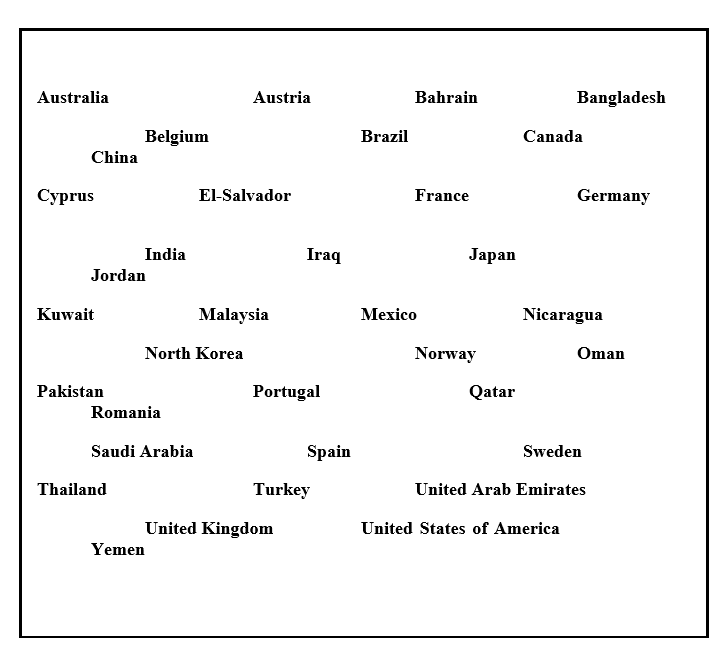


**Supplementary figure S1. Location of African in Diaspora included in the survey.** Africans in diaspora refers to those who went abroad for study or works, but not Africans born abroad.

1. Information from some of these sources may be unempirical, unverified and can be misleading thus creating infodemic, myths, misconceptions, misinformation and stigmatization (Islam *et al*. (2020). Am J Trop Med & Hyg, 103(4), 1621–1629, <https://doi.org/10.4269/ajtmh.20-0812>; Schmidt et al. (2020). PLoS ONE 15(12): e0244420. <https://doi.org/10.1371/journal.pone.0244420>; Ahinkorah et al. (2020). Front. Commun. 5:45, <https://doi.org/10.3389/fcomm.2020.00045>; Swire-Thompson & Lazer (2020). Ann Rev Pub Health, 41:433-451, <https://doi.org/10.1146/annurev-publhealth-040119-094127>). [↑](#footnote-ref-1)
